# Supplementary material for: Measuring agreement among several raters classifying subjects into one or more (hierarchical) categories: A generalization of Fleiss’ kappa
Source: Behav Res Methods. 2025 Sep 15;57(10):287. doi: 10.3758/s13428-025-02746-8 (PMC12436533; doi:10.3758/s13428-025-02746-8)
Supplement: Supplementary file 3 — (pdf 125 KB) [file 13428_2025_2746_MOESM3_ESM.pdf]

# Appendix A - Proof: The proposed $\kappa$ statistic is a generalization of Fleiss' kappa

Filip Moons

**Theorem 1.** *In case of equally weighted, mutually exclusive and non-hierarchical categories, the proposed kappa-statistic in Equation 8 reduces to the Fleiss' kappa.*

*Proof.* As the categories are mutually exclusive, we know that $\sum_c x_{ijc} = 1$ for every combination of $i$ and $j$, hence:

$$\sum_i \sum_c x_{ic} = \sum_i \sum_c \sum_j x_{ijc} = \sum_i \sum_j 1 = IJ. \tag{A.1}$$

Because the categories are equally weighted, we use the following formula from the main article:

$$\kappa = \frac{\sum\limits_c (Po_c - Pe_c)}{\sum\limits_c (1 - Pe_c)}.$$

First, we rewrite the denominator. Based on the formula of $Pe$ and (A.1) we get that:

$$\begin{aligned}
\sum_c (1 - Pe_c) &= \sum_c \left[ 1 - 2\left(\frac{\sum_i x_{ic}}{IJ}\right)^2 + 2\left(\frac{\sum_i x_{ic}}{IJ}\right) - 1 \right] \\
&= -2\sum_c \left(\frac{\sum_i x_{ic}}{IJ}\right)^2 + 2\sum_c \left(\frac{\sum_i x_{ic}}{IJ}\right) \\
&= -2\sum_c \left(\frac{\sum_i x_{ic}}{IJ}\right)^2 + 2\left(\frac{\sum_c \sum_i x_{ic}}{IJ}\right) \\
&= -2\sum_c \left(\frac{\sum_i x_{ic}}{IJ}\right)^2 + 2. \qquad \text{(A.2)}
\end{aligned}$$

Second, based on the formulas of $Po$ and $Pe$, the numerator equals:

$$\begin{aligned}
&\sum_c (Po_c - Pe_c) \\
&\quad = \sum_c \left[ \sum_i \frac{2x_{ic}^2 - 2Jx_{ic} + J^2 - J}{IJ(J-1)} - 2\left(\frac{\sum_i x_{ic}}{IJ}\right)^2 + 2\left(\frac{\sum_i x_{ic}}{IJ}\right) - 1 \right] \\
&\quad = \sum_c \sum_i \frac{2x_{ic}^2 - 2Jx_{ic} + J^2 - J}{IJ(J-1)} - 2\sum_c \left(\frac{\sum_i x_{ic}}{IJ}\right)^2 + 2\sum_c \left(\frac{\sum_i x_{ic}}{IJ}\right) - C,
\end{aligned}$$

applying (A.1):

$$\begin{aligned}
&= \frac{2(\sum_i \sum_c x_{ic}^2) - 2JIJ + CIJ^2 - CIJ}{IJ(J-1)} - 2\sum_c \left(\frac{\sum_i x_{ic}}{IJ}\right)^2 + 2 - C \\
&= \frac{2(\sum_i \sum_c x_{ic}^2) - 2IJ^2 + CIJ^2 - CIJ + 2IJ(J-1) - CIJ(J-1)}{IJ(J-1)} - 2\sum_c \left(\frac{\sum_i x_{ic}}{IJ}\right)^2 \\
&= \frac{2(\sum_i \sum_c x_{ic}^2) - 2IJ}{IJ(J-1)} - 2\sum_c \left(\frac{\sum_i x_{ic}}{IJ}\right)^2. \qquad \text{(A.3)}
\end{aligned}$$

Finally, we divide (A.3) by (A.2) and get the well-known Fleiss' kappa (see section 'Fleiss kappa' in the introduction):

$$\kappa = \frac{\dfrac{(\sum_i \sum_c x_{ic}^2) - IJ}{IJ(J-1)} - \displaystyle\sum_c \left( \frac{\sum_i x_{ic}}{IJ} \right)^2}{1 - \displaystyle\sum_c \left( \frac{\sum_i x_{ic}}{IJ} \right)^2}.$$

□
