# Supplementary material for: Measuring agreement among several raters classifying subjects into one or more (hierarchical) categories: A generalization of Fleiss’ kappa
Source: Behav Res Methods. 2025 Sep 15;57(10):287. doi: 10.3758/s13428-025-02746-8 (PMC12436533; doi:10.3758/s13428-025-02746-8)
Supplement: Supplementary file 6 — (pdf 2450 KB) [file 13428_2025_2746_MOESM6_ESM.pdf]

Student answers to sample question

S1 - Student 1

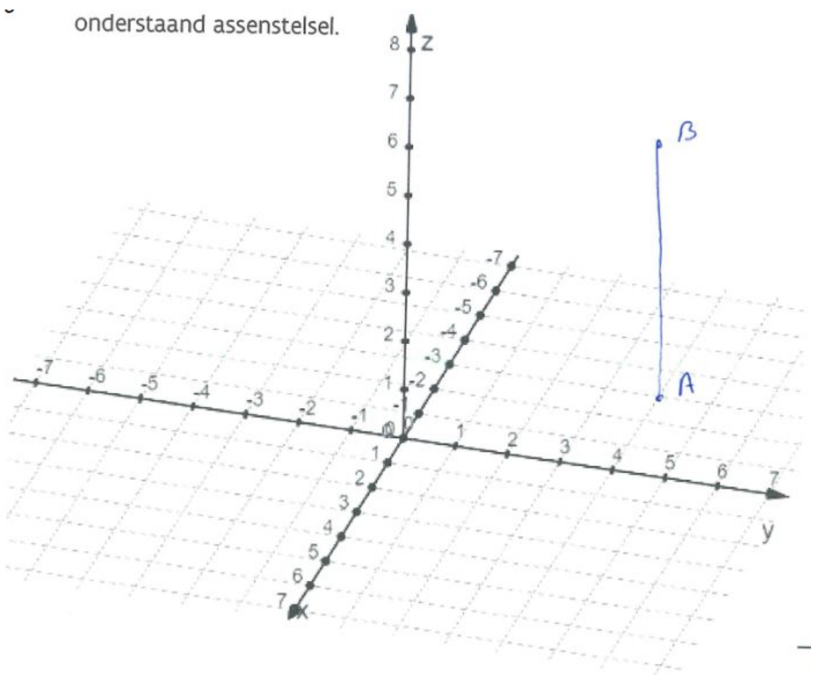

STUDENT ANSWERS TO SAMPLE QUESTION

S2 - Student 2

7) (/2,5) Teken het lijnstuk [AB] met $co(A) = (-3, 4, 0)$ en $co(B) = (-3, 4, 5)$

onderstaand assenstelsel.

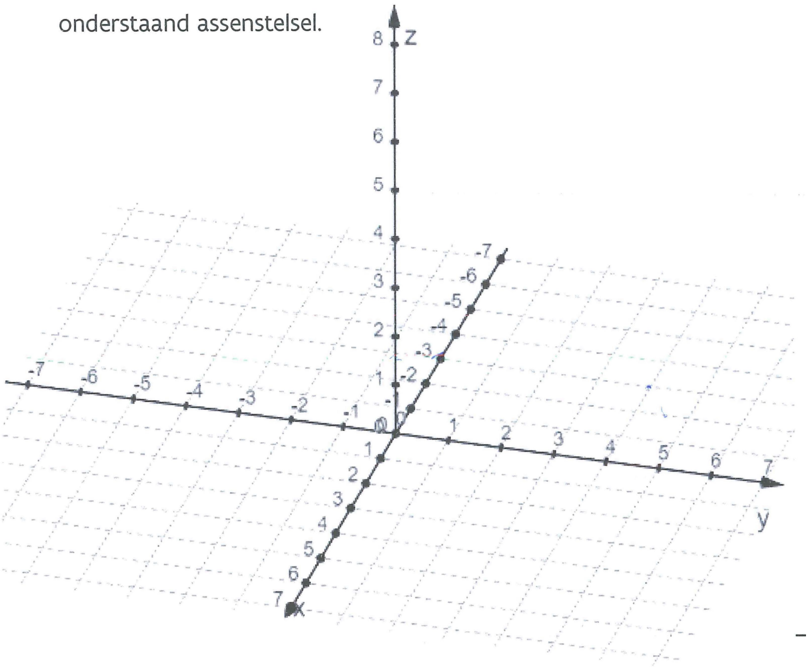

S3 - Student 3

onderstaand assenstelsel.

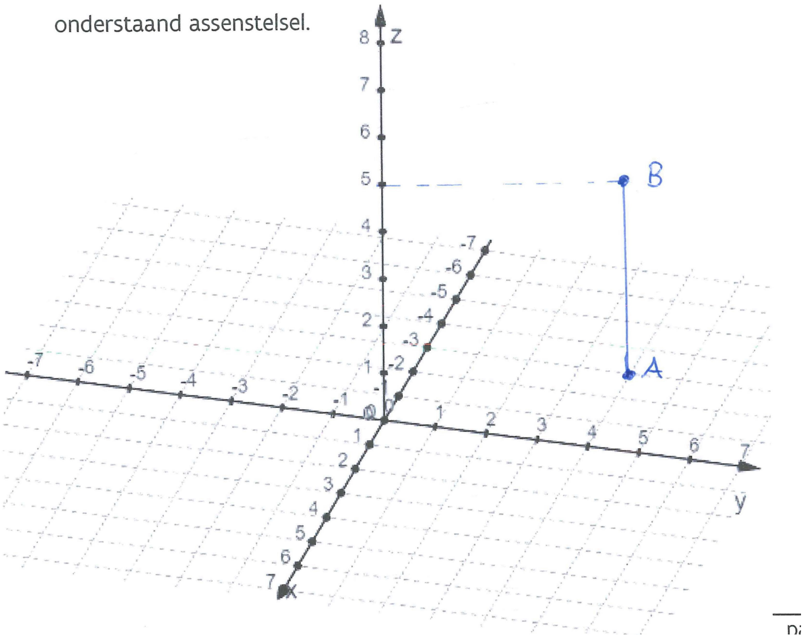

S4 - Student 4

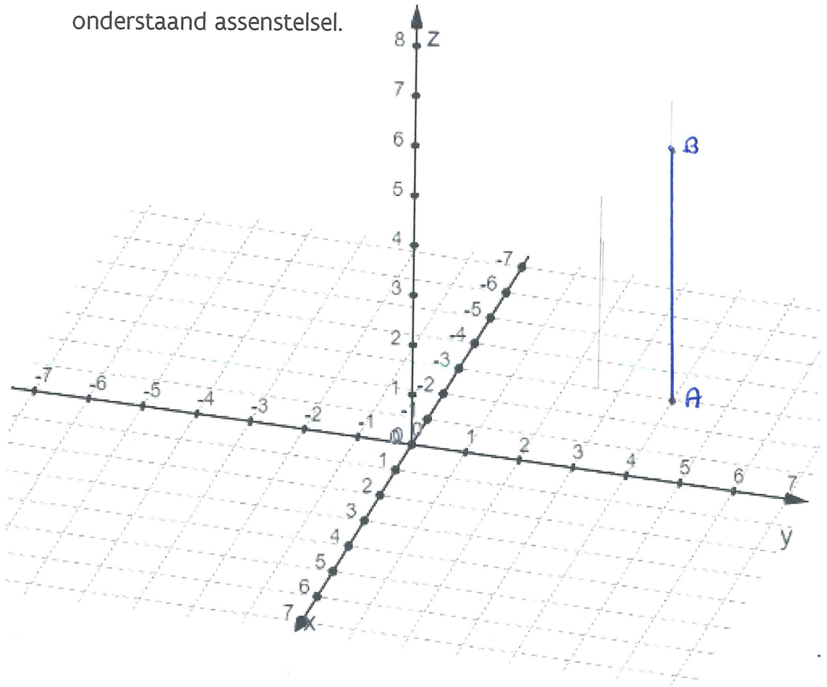

S5 - Student 5

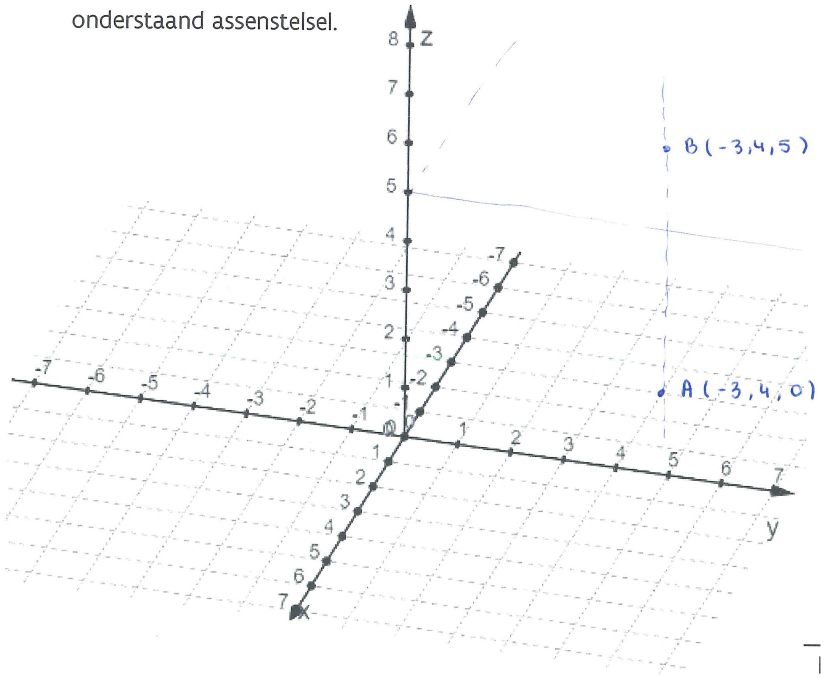

S6 - Student 6

7) (/2,5) Teken het lijnstuk [AB] met $co(A) = (-3,4,0)$ en $co(B) = (-3,4,5)$ in onderstaand assenstelsel.

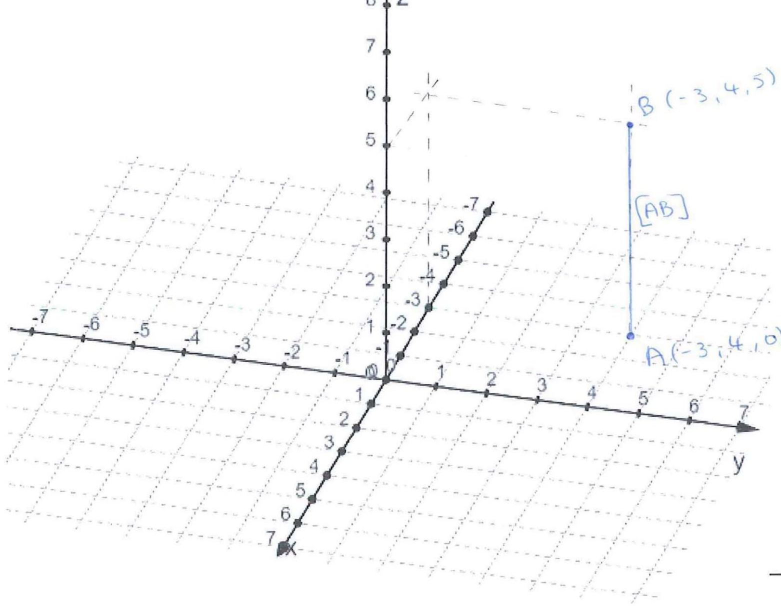

pagi
